# Comparison of skin dose in IMRT and VMAT with TrueBeam and Halcyon linear accelerator for whole breast irradiation

**DOI:** 10.1007/s13246-023-01373-x

**Published:** 2024-01-15

**Authors:** Jae Hyun Seok, So Hyun Ahn, Woo Sang Ahn, Dong Hyeok Choi, Seong Soo Shin, Wonsik Choi, In-hye Jung, Rena Lee, Jin Sung Kim

**Affiliations:** 1 Department of Integrative Medicine, Yonsei University College of Medicine, Seoul, Korea; 2 Medical Physics and Biomedical Engineering Lab (MPBEL), Yonsei University College of Medicine, Seoul, Korea; 3 Ewha Medical Research Institute, Ewha Womans University College of Medicine, Seoul, Korea; 4 Department of Radiation Oncology, Gangneung Asan Hospital, University of Ulsan College of Medicine, Gangneung, Korea; 5 Department of Medicine, Yonsei University College of Medicine, Seoul, Korea; 6 Department of Biomedical Engineering, Ewha Womans University, Seoul, Korea; 7 Department of Radiation Oncology, Yonsei Cancer Center, Yonsei University College of Medicine, Seoul, Korea; 8 Ewha Medical Artifical Intelligence Research Institute, Ewha Womans University College of Medicine, Seoul, Korea

**Keywords:** Halcyon, FFF beam, Breast cancer, Skin dose, O-ring-type linac

## Abstract

With the increasing use of flattening filter free (FFF) beams, it is important to evaluate the impact on the skin dose and target coverage of breast cancer treatments. This study aimed to compare skin doses of treatments using FFF and flattening filter (FF) beams for breast cancer. The study established treatment plans for left breast of an anthropomorphic phantom using Halcyon’s 6-MV FFF beam and TrueBeam’s 6-MV FF beam. Volumetric modulated arc therapy (VMAT) with varying numbers of arcs and intensity modulated radiation therapy (IMRT) were employed, and skin doses were measured at five points using Gafchromic EBT3 film. Each measurement was repeated three times, and averaged to reduce uncertainty. All plans were compared in terms of plan quality to ensure homogeneous target coverage. The study found that when using VMAT with two, four, and six arcs, in-field doses were 19%, 15%, and 6% higher, respectively, when using Halcyon compared to TrueBeam. Additionally, when using two arcs for VMAT, in-field doses were 10% and 15% higher compared to four and six arcs when using Halcyon. Finally, in-field dose from Halcyon using IMRT was about 1% higher than when using TrueBeam. Our research confirmed that when treating breast cancer with FFF beams, skin dose is higher than with traditional FF beams. Moreover, number of arcs used in VMAT treatment with FFF beams affects skin dose to the patient. To maintain a skin dose similar to that of FF beams when using Halcyon, it may be worth considering increasing the number of arcs.

## Introduction

Breast cancer is the most prevalent cancer among women in 159 out of 185 countries and ranks fifth among the leading causes of cancer-related mortality [1]. Treatment for breast cancer depends on the stage of the cancer, with breast-conserving surgery and whole-breast irradiation representing the most commonly utilized approach for early-stage breast cancer [2–3]. Whole-breast irradiation is a critical element of breast cancer treatment, as it has been shown to reduce the risk of cancer recurrence and improve patient survival rates [4].

The advent of the flattening filter free (FFF) beam has resulted in numerous considerations and changes in radiation therapy. Patients can be treated more efficiently using a high-dose rate, and the reduction of head scatter can minimize radiation exposure to healthy organs outside the treatment area [5]. The average energy of the FFF beam is low, as it experiences reduced beam-hardening effects, and high skin doses can be generated due to an increased presence of electron contamination [6]. However, it is important to consider that high skin doses can lead to erythema in patients with breast cancer, and efforts should be taken during treatment planning to minimize this possibility [7–8].

Given its imaging capabilities and workflow efficiency, linear accelerators that solely use FFF beams, such as Halcyon (Varian Medical Systems, Palo Alto, CA, USA), have lately been used for a wider range of treatment sites. Halcyon is an O-ring-type linear accelerator that exclusively employs a 6-MV FFF beam and is considered as an alternative to conventional C-arm-type linear accelerators. The unique design of the Halcyon accelerator, located within the bore, enables rotation speeds to be increased up to 4 rpm, without posing any collision risks [9]. Moreover, the Halcyon accelerator can deliver a high dose rate of up to 800 MU/min, enabling a reduction in treatment times due to the fast gantry rotation speed and high dose rate [10, 11].

Conventional C-arm-type linear accelerators, such as TrueBeam (Varian Medical Systems), are capable of selectively using both flattened-beam and FFF beam modes, making them suitable for treating skin areas such as in breast cancer, where flattened beams are usually employed. In contrast, Halcyon exclusively uses FFF beams, making it imperative to utilize FFF beams for all treatment sites. This study aimed to evaluate the increase in skin doses when breast cancer, should be cautioned to mitigate the risk of overdosing skin tissue, is treated with a FFF beam. Skin doses were compared and evaluated during breast irradiation using TrueBeam and Halcyon.

## Materials and methods

### VMAT and IMRT plans

**Fig. 1:**
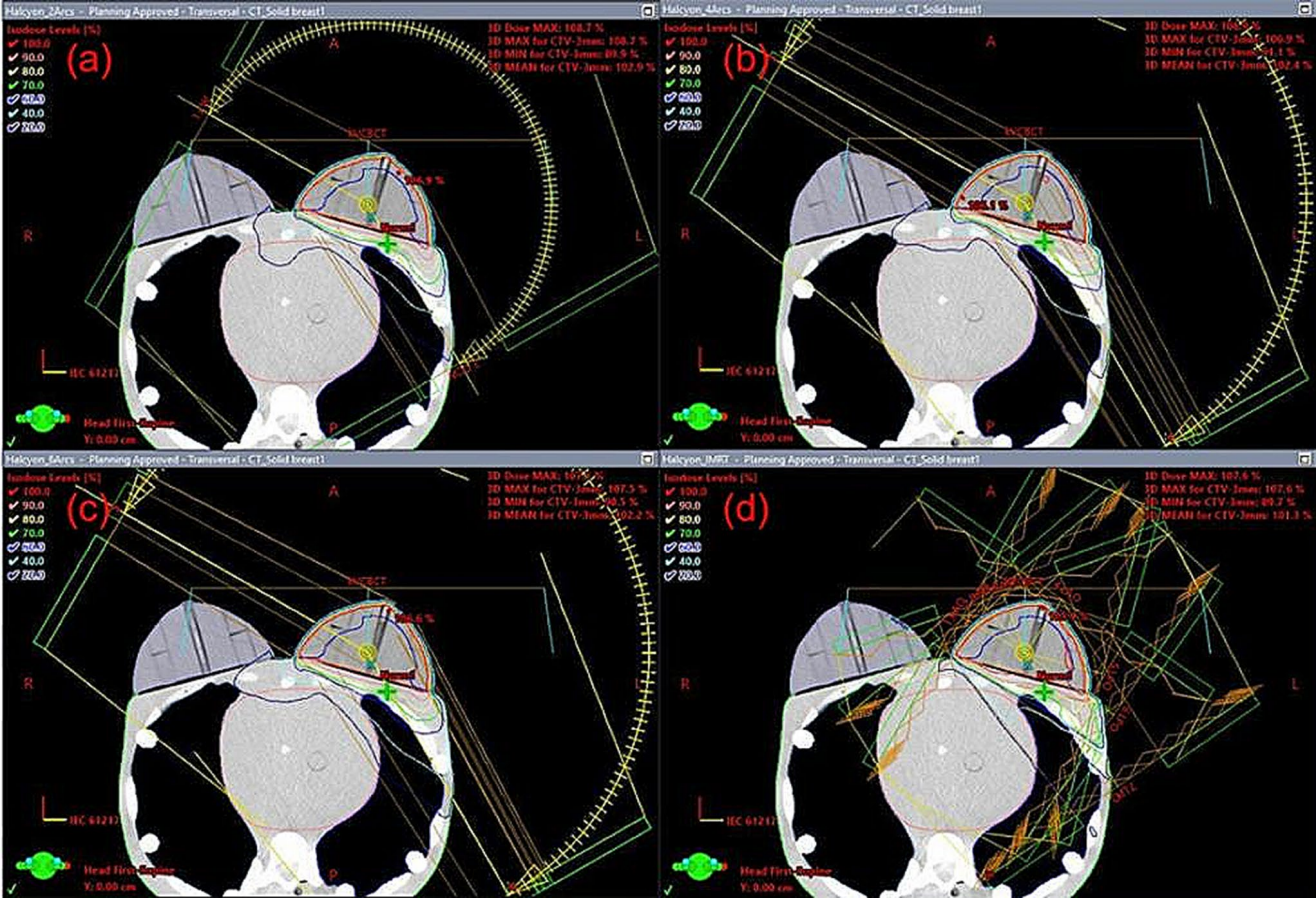
Dose distribution of **a** VMAT-2A, **b** VMAT-4A, **c** VMAT-6A, and **d** 7F-IMRT plans for Halcyon

To acquire the necessary data for our study, we obtained computed tomography (CT) images of the upper body of the RANDO phantom (CIRS Inc., Norfolk, VA, USA), a widely-used anthropomorphic phantom for radiation dosimetry measurements, using a Discovery RT scanner (GE Healthcare, Waukesha, WI, USA) with a tube voltage of 120 kVp, tube current of 450 mA, and slice thickness of 2.5 mm. Treatment plans were then created using the Eclipse treatment planning system version 16.1 with the AAA dose calculation algorithm from Varian Medical Systems for both the TrueBeam and Halcyon linear accelerators (linac). Four plans were designed for Halcyon with FFF beam and for TrueBeam with FF beam: intensity-modulated radiation therapy with seven fields (7F-IMRT) and volumetric modulated arc therapy with two arcs (VMAT-2A), four arcs (VMAT-4A) and six arcs (VMAT-6A), as shown in Fig. 1. The prescribed dose for the entire left breast was set at 200 cGy for all plans (Table 1).

**Table 1:** Summarized information about the breast cancer treatment plans used in this study

TPS	Linac	Plan	Beam type	Prescribed dose
Eclipse 16.1	Halcyon	VMAT-2A, VMAT-4A, VMAT-6A	FFF^a^	200 cGy
		7F-IMRT		
	TrueBeam	VMAT-2A, VMAT-4A, VMAT-6A	FF^b^	
		7F-IMRT		

^a^Flattening filter free beam

^b^Flattened beam

### Measurements

**Fig. 2:**
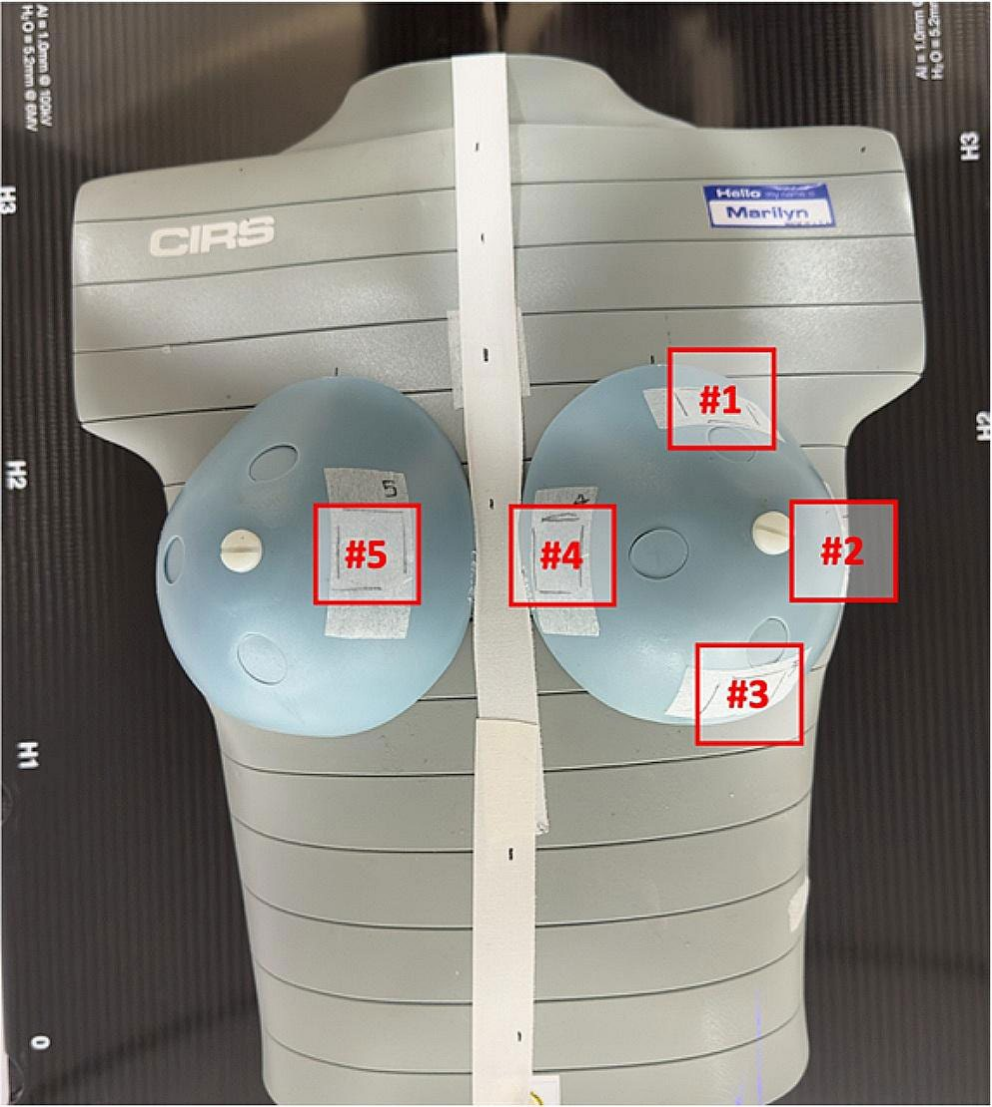
RANDO phantom setup showing measurement points indicated on the phantom

To measure the skin and in-field doses, Gafchromic EBT3 films with dimensions of 2 × 2 cm^2^ were placed at five different points. As shown in Fig. 2, four films were placed around the nipple of the left breast, which was the target area, at positions superior #1, to the right #2, inferior #3, and to the left #4, while the fifth film was placed on the opposite breast #5 to measure the out-field dose. Each measurement point is marked to position the film in the correct position. Each measurement was repeated three times for each treatment plan. The locations of the films are shown in Fig. 2 for reference.

**Fig. 3:**
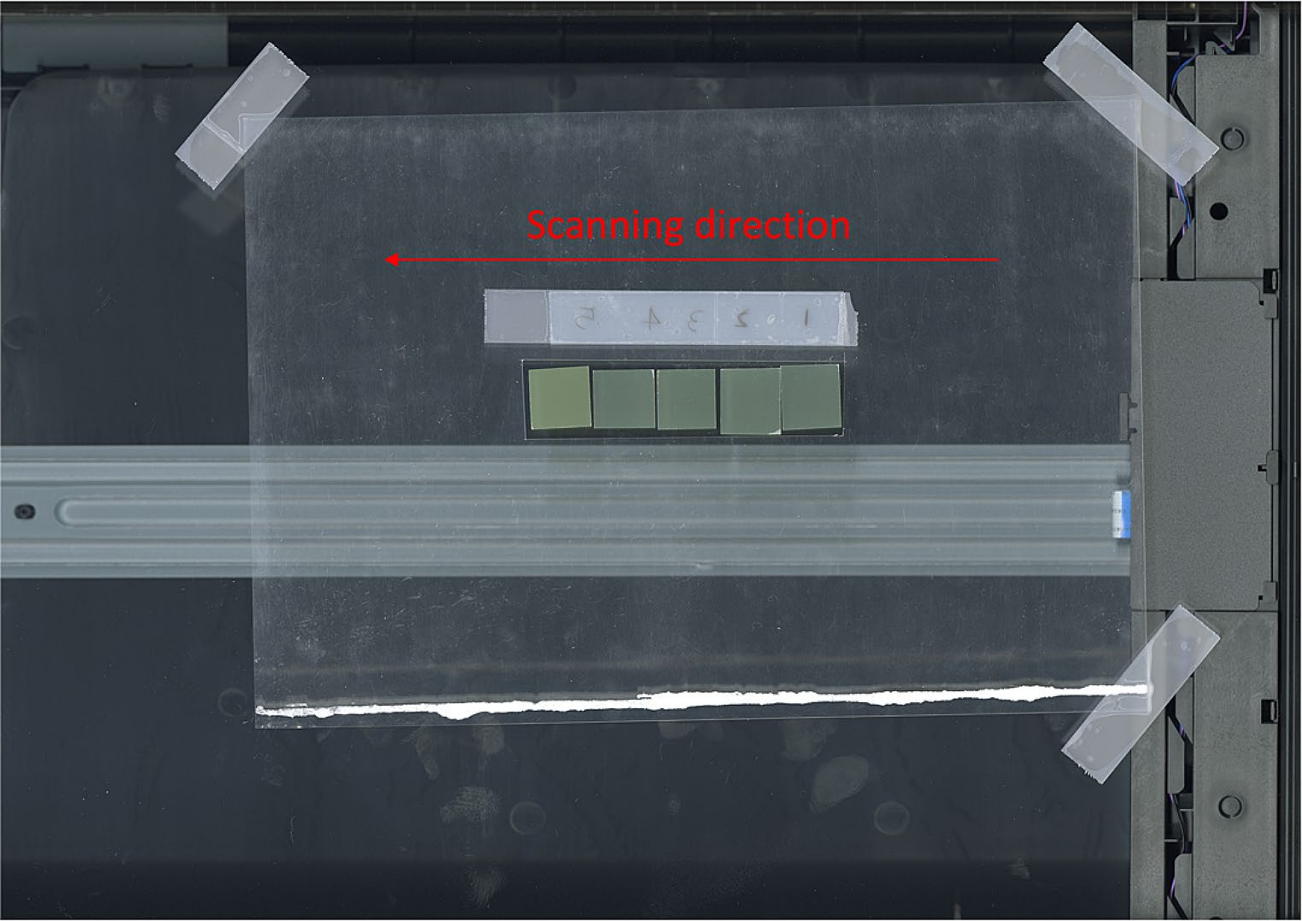
EBT3 film positioned in frame attached at center of the scanning bed

The Gafchromic EBT3 film was scanned using an Epson Expression 12000XL scanner (Seiko Epson Corporation, Nagano, Japan) in reflective mode with a resolution of 600 dpi and a 16-bit color scale. The red channel of the RGB color image was analyzed using ImageJ (Wayne Rasband, National Institute of Health, USA). To ensure accuracy, three measurements were taken and then averaged, and a calibration curve was used to convert the scan values into dose values. It has been reported that flatbed document scanners exhibit a significant dependence in lateral response as the Region of Interest (ROI) approaches the horizontal edges of the scanning bed [12], however no significant change in sensitivity of transmission pixels was observed depending on the scanning direction. To ensure reproducible scanning conditions, a frame was attached to the center of the scanning bed to maintain the ROI and film position along the scanning direction, as shown in Fig. 3. The film doses were calibrated using ion chamber (Farmer-type; active volume, 0.6 cc) values under the same conditions. The calibration was done at 6 MV, with a source-to-detector distance of 100 cm and a depth of 5 cm in Solid Water. To measure doses, a 10 × 10 cm^2^ field was used and 20 measurements were taken between 6 MU and 500 MU.

### Evaluation

In this study, we compared the skin doses measured using two types of radiation beams: flattened filter (FF) beams (TrueBeam) and flattening filter-free (FFF) beams (Halcyon). First, we evaluated the differences between the doses measured using VMAT and IMRT plans on both machines, at each point and total difference between in-field dose. Secondly, we compared the VMAT-2A plan on Halcyon to VMAT-4A and VMAT-6A plan on Halcyon, at each point and averaged difference between in-field dose. To ensure accurate results, we repeated the measurements three times and calculated the average value to minimize potential errors.

## Results

### Plan evaluation

Table 2 shows the plan quality using HI and CI indexes. HI indicates the index of homogeneity, and CI indicates the index of conformity. The HI was calculated using the following equation:

1 $$ HI= \frac{{D}_{5\%}-{D}_{95\%}}{{D}_{mean}}, $$

where D_5%_ and D_95%_ are the absolute doses covering 5% and 95% of the target volume, respectively. CI was calculated using the following equation:

2 $$ CI= \frac{{{TV}_{PTV}}^{2}}{(TV \times PIV)},$$

where $$ {TV}_{PTV}$$ is the planning target volume (PTV) receiving 95% of the prescribed dose, TV is the total volume of the CTV, and PIV is the total volume covered by the prescribed 95% isodose.

Table 2 shows information about the dose volume histogram (DVH) of organs at risk (OARs). The V_40Gy_ and V_30Gy_, which indicate DVHs in general, were excluded from this study. The D_mean_ was analyzed for the DVHs of the lung, ipsilateral lung, the heart, and the contralateral breast.

**Table 2:** Dosimetric parameters of target and OARs for IMRT and VMAT plans on the TrueBeam and Halcyon machine

Linac	Plan	Type of beam	Target		OAR				MU
					D_mean_ [cGy]				
			HI	CI	Whole lung	Ipsilateral lung	Heart	Contralateral breast	
Halcyon	VMAT-2A	FFF	0.052	0.869	323.4	510.4	391.9	499.9	531.1
	VMAT-4A		0.044	0.874	345.8	558.6	373.8	485.5	511.0
	VMAT-6A		0.041	0.872	351.6	567.8	381.7	491.3	531.5
	7F-IMRT		0.034	0.826	212.2	383.0	264.1	245.5	1040.4
TrueBeam	VMAT-2A	FF	0.042	0.880	321.7	499.9	387.0	538.6	509.8
	VMAT-4A		0.034	0.881	349.7	551.3	374.9	518.1	537.4
	VMAT-6A		0.035	0.880	352.7	565.9	375.3	508.2	507.9
	7F-IMRT		0.031	0.831	214.8	382.0	287.1	258.4	968.7
Average			0.039	0.864	309.0	502.4	354.5	443.2	642.2
STDEV			0.007	0.022	60.18	78.09	49.47	119.2	224.7

### Dose measurements

Table 3 shows the measured data for a single fraction at each point of VMAT-2A, VMAT-4A, VMAT-6A and 7F-IMRT plan using TrueBeam. To analyze the uncertainty, measurements were taken three times, and the standard deviation of the measured values was analyzed.

**Table 3:** Measured dose of VMAT-2A, VMAT-4A, VMAT-6A and 7F-IMRT plans of TrueBeam at each measurement point

Position	Plan			
	VMAT-2A	VMAT-4A	VMAT-6A	7F-IMRT
	Dose [cGy]	Dose [cGy]	Dose [cGy]	Dose [cGy]
1 (superior)	125.18 ± 11.29	125.26 ± 6.88	127.05 ± 5.86	134.72 ± 10.03
2 (right)	155.66 ± 16.13	138.99 ± 5.51	140.95 ± 5.29	144.30 ± 13.76
3 (inferior)	114.76 ± 2.61	111.87 ± 7.77	119.72 ± 14.75	115.00 ± 9.41
4 (left)	147.24 ± 9.59	138.00 ± 4.11	148.61 ± 10.10	145.22 ± 5.97
5 (opposite side)	47.84 ± 2.42	46.75 ± 3.41	43.80 ± 4.97	93.34 ± 6.57

Table 4 shows the measured data for a single fraction at each point of VMAT-2A, VMAT-4A, VMAT-6A and 7F-IMRT plan using Halcyon. To analyze the uncertainty, measurements were taken three times, and the standard deviation of the measured values was analyzed.

**Table 4:** Measured dose of VMAT-2A, VMAT-4A, VMAT-6A and 7F-IMRT plans of Halcyon at each measurement point

Position	Plan			
	VMAT-2A	VMAT-4A	VMAT-6A	7F-IMRT
	Dose [cGy]	Dose [cGy]	Dose [cGy]	Dose [cGy]
1 (superior)	183.75 ± 4.48	167.11$\pm9$0.63	142.84 ± 4.70	160.09 ± 12.09
2 (right)	151.23 ± 10.88	147.90 ± 7.86	141.63 ± 3.83	160.65 ± 6.75
3 (inferior)	148.70 ± 10.20	126.09 ± 6.66	132.24 ± 2.55	108.33 ± 6.78
4 (left)	174.64 ± 7.83	154.88 ± 6.13	149.55 ± 3.52	140.70 ± 8.51
5 (opposite side)	61.71 ± 3.34	48.93 ± 4.02	47.54 ± 4.29	92.59 ± 3.00

**Fig. 4:**
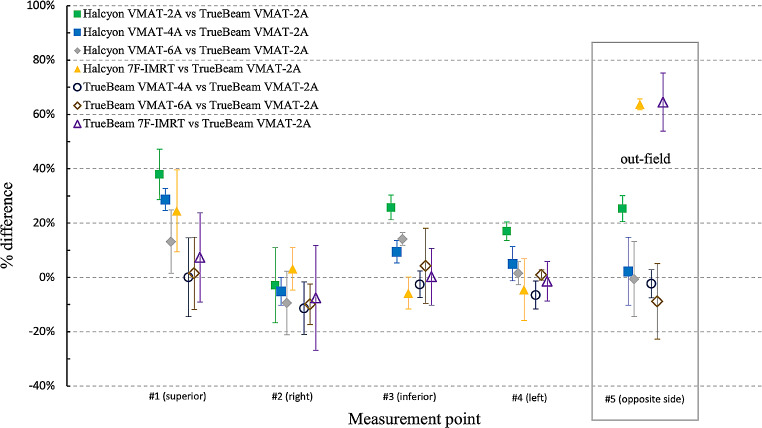
Comparison of the measured doses at each measurement point between VMAT-2A plan for TrueBeam and different plans

Figure 4 shows the percent differences for a single fraction at each point of the VMAT-2A, VMAT-4A, VMAT-6A and 7F-IMRT plans using Halcyon and TrueBeam compared with the VMAT-2A plan using TrueBeam. The IMRT plans using Halcyon and TrueBeam delivered significantly higher doses than the VMAT plans using Halcyon and TrueBeam in the out-field point.

Table 5 shows absolute dose differences and percent differences for a single fraction in the VMAT plans using Halcyon and TrueBeam. Most of the in-field points using Halcyon for the VMAT plans show higher skin doses compared with the VMAT plans using TrueBeam. The averaged dose differences in the in-field points were measured up to 28.86 cGy. The absolute dose differences of the out-field points were 2.19–13.87 cGy.

**Table 5:** Absolute dose differences and percent differences of VMAT-2A, VMAT-4A, VMAT-6A plans for Halcyon and TrueBeam; side-by-side comparison

Position	Plan					
	VMAT-2A (Hal^a^ vs. TB^b^)		VMAT-4A (Hal^a^ vs. TB^b^)		VMAT-6A (Hal^a^ vs. TB^b^)	
	Diff. (%)	Dose diff. [cGy]	Diff. (%)	Dose diff. [cGy]	Diff. (%)	Dose diff. [cGy]
1 (superior)	38%	58.57 ± 0.09	29%	41.85 ± 0.11	12%	15.79 ± 0.04
2 (right)	-3%	-4.44 ± 0.14	6%	8.91 ± 0.06	0%	0.68 ± 0.06
3 (inferior)	26%	33.95 ± 0.05	12%	14.22 ± 0.07	10%	12.52 ± 0.11
4 (left)	17%	27.37 ± 0.03	12%	16.87 ± 0.06	1%	0.94 ± 0.04
In-field average	19%	28.86 ± 0.03	15%	20.46 ± 0.05	6%	7.48 ± 0.04
5 (opposite side)	25%	13.87 ± 0.05	5%	2.19 ± 0.15	8%	3.74 ± 0.16

^a^Halcyon

^b^TrueBeam

Table 6 shows absolute dose differences and percent dose differences for a single fraction between Halcyon and TrueBeam measurements. Most of the in-field points for the VMAT plans using Halcyon showed higher skin doses compared with the IMRT plans using TrueBeam. The averaged dose differences of VMAT plans using Halcyon compared with the IMRT plans using TrueBeam for the in-field points were measured up to 29.77 cGy. Out-field point absolute dose differences were − 45.80 to − 31.63 cGy. The averaged dose differences between IMRT plans using Halcyon or TrueBeam were 7.63 cGy at in-field points and − 0.75 cGy at the out-field point.

**Table 6:** Absolute dose differences and percent differences of Halcyon treatment plans compared to 7F-IMRT plan for TrueBeam.

Position	Plan							
	Hal VMAT-2A vs. TB 7F-IMRT		Hal VMAT-4A vs. TB 7F-IMRT		Hal VMAT-6A vs. TB 7F-IMRT		Hal 7F-IMRT vs. TB 7F-IMRT	
	Diff. (%)	Dose diff. [cGy]	Diff. (%)	Dose diff. [cGy]	Diff. (%)	Dose diff. [cGy]	Diff. (%)	Dose diff. [cGy]
1 (superior)	31%	49.03 ± 0.07	21%	32.39 ± 0.13	6%	8.12 ± 0.05	17%	25.37 ± 0.05
2 (right)	5%	6.93 ± 0.08	2%	3.60 ± 0.15	-2%	-2.67 ± 0.10	11%	16.35 ± 0.13
3 (inferior)	26%	33.71 ± 0.15	9%	11.09 ± 0.12	14%	17.24 ± 0.09	-6%	-6.67 ± 0.11
4 (left)	18%	29.41 ± 0.07	6%	9.65 ± 0.08	3%	4.33 ± 0.05	-3%	-4.53 ± 0.04
In-field average	20%	29.77 ± 0.08	10%	14.18 ± 0.11	5%	6.76 ± 0.06	5%	7.63 ± 0.10
5 (opposite side)	-41%	-31.63 ± 0.10	-62%	-44.41 ± 0.03	-65%	-45.80 ± 0.06	-1%	-0.75 ± 0.06

## Discussion

Some previous studies have already demonstrated that increased skin doses occur when using FFF beams by plan study, Monte-Carlo simulations, and retrospective studies. Most planned and retrospective studies were conducted through treatment planning system, focusing on DVH or target coverage rather than skin dose [13, 14].

**Table 7:** Percentage of mean measured dose relative to calculated dose at in-field points for each treatment plan

Linac	Plan				
	VMAT-2A	VMAT-4A	VMAT-6A	7F-IMRT	Total
	% to TPS	% to TPS	% to TPS	% to TPS	% to TPS
Halcyon	112.2% ± 8.7%	101.0% ± 8.9%	98.6% ± 5.7%	92.4% ± 14.1%	101.2% ± 11.9%
TrueBeam	94.4% ± 7.9%	90.4% ± 5.8%	95.1% ± 7.7%	89.8% ± 7.0%	92.3% ± 7.3%
Diff	17.8%	10.6%	3.5%	2.6%	8.9%

Halcyon has been widely adopted for its advantages, including faster treatment times facilitated by its high gantry rotation speed and high dose rate, and it is utilized for various treatment sites [10, 11]. In contrast to conventional breast cancer treatment using FF beams, Halcyon can be exclusively utilized with FFF beams for treatment. It has been previously documented that the use of Halcyon’s FFF beam in breast cancer treatment results in an increased skin dose compared to using FF beams [15, 16]. Our results are consistent with these findings. We proceed a total of 24 measurements with 12 measurements each for Halcyon and TrueBeam. The average of measured dose at in-field points were 19%, 15%, 6% and 5% higher, respectively, when using Halcyon compared to TrueBeam using VMAT-2A, VMAT-4A, VMAT-6A and 7F-IMRT plan. Similar phantom measurements made by O’Grady et al., with optically stimulated luminescence detectors (OSLD) using plan with field-in-field technique and irregular surface compensator technique, the average superficial dose measured as 70% ± 1.3% with Halcyon, which demonstrates an increase of approximately 14% with respect to TrueBeam [16]. As shown in Table 7, in our study the percentage of mean value of measured dose to calculated dose were 92.3% ± 7.3% with TrueBeam and 101.2% ± 11.9% with Halcyon, which demonstrates and increase of approximately 9% with respect to TrueBeam. Despite the use of different planning techniques, forward planning and inverse planning, may cause discrepancies in amount of increase, the tendency of Halcyon to exhibit a higher skin dose was consistently observed.

**Fig. 5:**
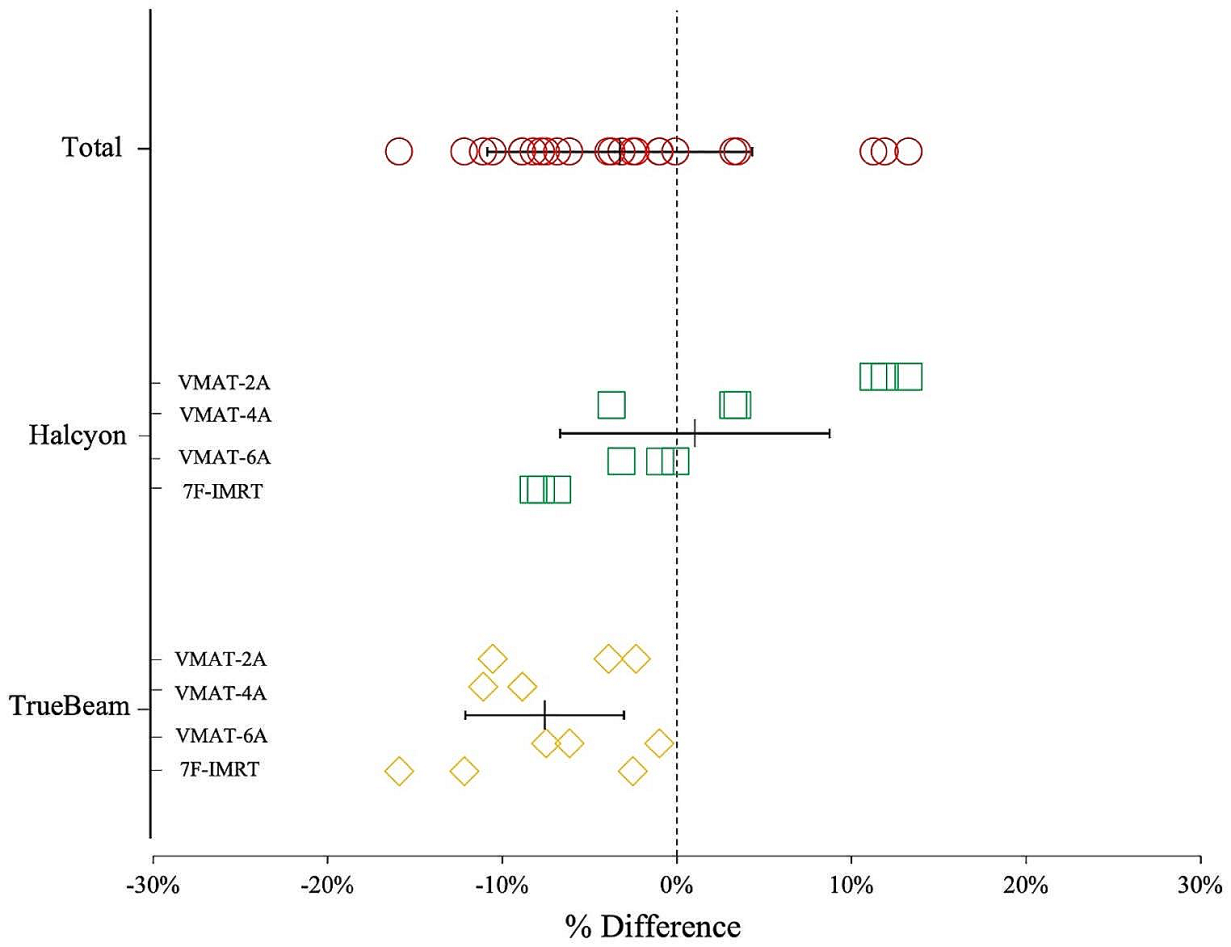
Percentage difference between the mean of measured dose and calculated dose from TPS for different linacs

As can be seen in Table 2, there is no discernible difference in the plan quality of the target. By contrast, the percentage difference between the mean of measurement dose and calculated dose from TPS showed a total of -3.3% ± 7.6%, with 1.0% ± 7.7% in Halcyon and − 7.6% ± 4.5% in TrueBeam, as showed in Fig. 5. We assumed that the reason for these difference could be caused by the difficulty in accurately defining the ROI at the skin of the phantom, which is measurement point where the film was attached, in the TPS could have introduced errors. Interestingly, an increase in the number of arcs in the VMAT plan using Halcyon showed a tendency to reduce the skin dose, as shown in Table 8 and Fig. 5, narrowing the difference with using TrueBeam, as shown in Table 7. Considering these results, increasing the number of arcs in VMAT could be considered to achieve a skin dose similar to that of FF beams. However, increasing the number of arcs would lead to an increase in treatment time by approximately one minute per arc. It is recommended to carefully consider whether increasing the number of arcs is feasible in the clinical setting, taking into account the increase in treatment time at each facility. Lonski et al. used an ion chamber to measure skin dose for various field sizes ranging from 4 × 4 to 40 × 40 cm^2^ [17]. The authors confirmed that the 6 FFF beam produces a higher skin dose than the 6 FF beam, and the 10 FFF beam produces a lower skin dose than the 10 FF beam. Therefore, the result shown in our study that FFF beams have a higher skin dose than FF beams as shown in Table 8 may not apply to facilities using higher energies 10 MV or above.

**Table 8:** Absolute and percent dose differences of VMAT-2A plan for Halcyon compared to VMAT-4A and VMAT-6A plans for Halcyon

Position	Plan			
	VMAT-2A vs. VMAT-4A		VMAT-2A vs. VMAT-6A	
	Diff. (%)	Dose diff. [cGy]	Diff. (%)	Dose diff. [cGy]
1 (superior)	9%	16.64	25%	40.91
2 (right)	2%	3.33	7%	9.59
3 (inferior)	16%	22.61	12%	16.46
4 (left)	12%	19.76	15%	25.09
In-field average	10%	15.59	15%	23.01
5 (opposite side)	23%	12.78	26%	14.17

In recent years, inverse planning techniques such as IMRT and VMAT have become the predominant methods, replacing fluence-based planning approaches like the irregular surface compensator tool or field-in-field (FiF) plan [18]. Therefore, the objective of this study was to investigate the differences in skin doses when employing FFF and FF beams, in conjunction with various planning methodologies. A limitation of this study is that it was conducted using a solid phantom, which lacks the ability to accurately reflect the dynamic nature of the breast composed of soft tissue, including overlapping and tissue movement that occurs in clinical practice. Future studies should incorporate a wider range of plans and phantoms to better simulate clinical practices and include statistical tests to provide more precise and robust results.

## Conclusion

In this study, we successfully measured the amount of radiation that the skin receives during whole-breast irradiation treatments using both Halcyon and TrueBeam. The results showed that the dose at specific points was higher when the Halcyon FFF beam was used compared to the TrueBeam FF beam. Also, the amount of radiation that the skin received decreased as the number of arcs in the VMAT plan increased for the Halcyon FFF beam. It is important to consider these effects when planning breast cancer treatments using FFF beams.
