# Correction to: Comparison of skin dose in IMRT and VMAT with TrueBeam and Halcyon linear accelerator for whole breast irradiation

**DOI:** 10.1007/s13246-024-01395-z

**Published:** 2024-02-05

**Authors:** Jae Hyun Seok, So Hyun Ahn, Woo Sang Ahn, Dong Hyeok Choi, Seong Soo Shin, Wonsik Choi, In-hye Jung, Rena Lee, Jin Sung Kim

**Affiliations:** 1 Department of Integrative Medicine, Yonsei University College of Medicine, Seoul, Korea; 2 Medical Physics and Biomedical Engineering Lab (MPBEL), Yonsei University College of Medicine, Seoul, Korea; 3 Ewha Medical Research Institute, Ewha Womans University College of Medicine, Seoul, Korea; 4 Ewha Medical Artifical Intelligence Research Institute, Ewha Womans University College of Medicine, Seoul, Korea; 5 Department of Radiation Oncology, Gangneung Asan Hospital, University of Ulsan College of Medicine, Gangneung, Korea; 6 Department of Medicine, Yonsei University College of Medicine, Seoul, Korea; 7 Department of Biomedical Engineering, Ewha Womans University, Seoul, Korea; 8 Department of Radiation Oncology, Yonsei Cancer Center, Yonsei University College of Medicine, Seoul, Korea

**Physical and Engineering Sciences in Medicine**

10.1007/s13246-023-01373-x

In this article the author’s name Woo Sang Ahn was incorrectly written as Sang Woo Ahn.

The original article has been corrected.

